# SARS-CoV-2 variants in severely symptomatic and deceased persons who had been vaccinated against COVID-19 in São Paulo, Brazil

**DOI:** 10.26633/RPSP.2021.126

**Published:** 2021-10-25

**Authors:** Karoline Rodrigues Campos, Cláudio Tavares Sacchi, Adriano Abbud, Adele Caterino-de-Araujo

**Affiliations:** 1 Laboratório Estratégico Centro de Respostas Rápidas Instituto Adolfo Lutz São Paulo, SP Brasil Laboratório Estratégico, Centro de Respostas Rápidas, Instituto Adolfo Lutz, São Paulo, SP, Brasil.; 2 Centro de Respostas Rápidas Instituto Adolfo Lutz São Paulo, SP Brasil Centro de Respostas Rápidas, Instituto Adolfo Lutz, São Paulo, SP, Brasil.; 3 Centro de Imunologia Instituto Adolfo Lutz São Paulo, SP Brasil Centro de Imunologia, Instituto Adolfo Lutz, São Paulo, SP, Brasil

**Keywords:** Betacoronavirus, coronavirus infections, vaccines, cause of death, risk groups, Brazil, Betacoronavirus, infecciones por coronavirus, vacunas, causas de muerte, grupos de riesgo, Brasil, Betacoronavirus, infecções por coronavirus, vacinas, causas de morte, grupos de risco, Brasil

## Abstract

COVID-19 vaccination began in São Paulo, Brazil in January 2021, first targeting healthcare workers (HCWs) and the elderly, using the CoronaVac vaccine (Sinovac/Butantan) and subsequently the Oxford/AstraZeneca (ChAdOx1) vaccine (AstraZeneca/FIOCRUZ-RJ). Studies on such vaccines have shown efficacy in preventing severe cases and deaths, but there is a lack of information regarding their effectiveness. This manuscript presents data from the Instituto Adolfo Lutz (IAL), a public health laboratory located in São Paulo City that receives samples from 17 Regional Health Departments under the Secretary of Health of São Paulo, for SARS-CoV-2 genomic surveillance. Through May 15, 2021 IAL received 20 samples for analysis from COVID-19 vaccinated individuals who needed hospitalization and/or died from COVID-19. Next-generation sequencing was performed on an Ion Torrent S5 platform using the AmpliSeq™ SARS-CoV-2 kit. Almost all cases were vaccinated with CoronaVac and presented the gamma variant of concern (VOC). Cases of death were observed mostly in the elderly in nursing homes, and severe cases in younger frontline HCWs. This data confirmed that the SARS-CoV-2 gamma variant is highly transmissible, severe, and lethal for COVID-19 in these groups of individuals, thereby highlighting the importance of continuous vaccination and non-pharmacological prevention measures to avoid virus dissemination and the emergence of new VOCs.

## RESUMEN

La vacunación contra la COVID-19 empezó en São Paulo (Brasil) en enero del 2021 con los trabajadores de atención de salud (personal de salud) y las personas mayores, empleando la vacuna de CoronaVac (Sinovac/Butantan) y posteriormente la vacuna de Oxford/AstraZeneca (ChAdOx1) (AstraZeneca/FIOCRUZ-RJ). Los estudios sobre estas vacunas han mostrado su eficacia en la prevención de los casos graves y las muertes, pero existe falta de información con respecto a su efectividad. En este artículo se presentan datos del Instituto Adolfo Lutz (IAL), un laboratorio de salud pública ubicado en la ciudad de São Paulo que recibe muestras de 17 departamentos regionales de salud bajo la Secretaría de Salud de São Paulo, relativos a la vigilancia genómica del SARS-CoV-2. Hasta el 15 de mayo del 2021, el IAL había recibido 20 muestras para su análisis de personas vacunadas contra la COVID-19 que necesitaron hospitalización o murieron a causa de esta enfermedad. Se realizó una secuenciación de nueva generación en una plataforma Ion Torrent S5 mediante el kit para el SARS-CoV-2 AmpliSeq™. Casi todos los pacientes se habían vacunado con CoronaVac y presentaban la variante de preocupación gamma. Se observaron muertes principalmente de personas mayores en residencias y casos graves en personal de salud más joven de primera línea. Estos datos confirmaron que la variante gamma del SARS-CoV-2 es sumamente transmisible, grave y letal para la COVID-19 entre estos grupos y destacan la importancia de continuar con la vacunación y las medidas preventivas no farmacológicas para evitar la propagación del virus y la aparición de nuevas variantes de preocupación.

## RESUMO

A vacinação contra a COVID-19 começou em São Paulo, Brasil, em janeiro de 2021, primeiramente dirigida a profissionais da saúde e idosos, utilizando a vacina CoronaVac (Sinovac/Butantan), e posteriormente a vacina Oxford/AstraZeneca (ChAdOx1) (AstraZeneca/Fiocruz-RJ). Os estudos sobre tais vacinas revelaram eficácia na prevenção de casos graves e mortes, mas há falta de informação em relação à sua efetividade. Este manuscrito apresenta dados do Instituto Adolfo Lutz (IAL), um laboratório de saúde pública localizado no município de São Paulo, que recebe amostras de 17 Departamentos Regionais de Saúde da Secretaria Estadual de Saúde de São Paulo para vigilância genômica do SARS-CoV-2. Até 15 de maio de 2021, o IAL recebeu 20 amostras para análise de indivíduos vacinados contra a COVID-19 que necessitaram de hospitalização e/ou morreram por COVID-19. O sequenciamento de nova geração foi realizado em plataforma Torrente de íon S5, utilizando o kit AmpliSeq™ SARS-CoV-2. Quase todos os casos foram vacinados com CoronaVac e apresentaram a variante de preocupação (VOC) gama. Os óbitos foram observados principalmente nos idosos de casas de repouso, e os casos graves em profissionais de saúde mais jovens da linha de frente. Esses dados confirmaram que a variante SARS-CoV-2 gama é altamente transmissível, grave e letal para COVID-19 nesses grupos de indivíduos, destacando, assim, a importância da vacinação contínua e de medidas preventivas não farmacológicas para evitar a disseminação viral e o surgimento de novas VOC.

The COVID-19 outbreak in Brazil has raised serious concerns since the end of 2020, when the second wave of the new SARS-CoV-2 lineage (previously named P.1, and now gamma) emerged in Manaus and spread rapidly nationwide (1). Brazil ranks third in confirmed cases and second in deaths worldwide (2), and the state of São Paulo accounts for the highest number of cases (more than 4 million), with over 139 000 deaths until August 1, 2021 (3).

In Brazil, COVID-19 vaccination started in São Paulo on January 17, 2021, using the CoronaVac inactivated vaccine (Sinovac/Butantan) and subsequently with the Oxford/AstraZeneca adenovirus vector vaccine (ChAdOx1) (AstraZeneca/FIOCRUZ-RJ). Brazil started vaccinating the population according to priority groups, first targeting healthcare workers (HCWs) and the elderly. By May 15, 13 236 339 individuals (above 60 years of age) and HCWs had been vaccinated in São Paulo (9 133 541 with one dose and 4 852 086 with two doses, representing 20.4% and 10.8%, respectively, of the state population), and CoronaVac accounted for 67.33% and ChAdOx1 for 32.67% of these vaccines (4). It should be noted that in São Paulo, until January 26, priority groups (HCWs and institutionalized elderly people) received the CoronaVac vaccine, and afterwards, the ChAdOx1 vaccine, depending on their availability at the immunization site.

Studies on vaccine efficacy (pivotal trials) showed a reduction in symptomatic cases. CoronaVac applied in Brazilian HCWs had 50.7% of overall efficacy, and 83.7% for moderate cases (5); ChAdOx1 applied in different populations in the UK, Brazil, and South Africa showed an overall efficacy of 66.7%, with differences depending on the interval between the two standard doses (55.1% efficacy after an interval of less than 6 weeks, and 81.3% after an interval of more than 12 weeks) (6). Although the number of severe cases and deaths made it statistically impossible to evaluate these endpoints, only one death by COVID-19 was reported for the phase III trial using CoronaVac, while only two severe cases, including one death, was found in the ChAdOx1 trial.

Recently, research on the effectiveness of CoronaVac was conducted at Hospital das Clínicas, São Paulo City, in a cohort of more than 21 000 HCWs who received their first dose on January 18–21 and the second dose on February, 14–16, 2021. The estimated effectiveness was of 50.7% and 51.8% at two and three weeks after the second dose, respectively. Genomic sequencing of 142 samples found that 47% of the SARS-CoV-2 variants of concern (VOCs) were mostly gamma (7). However, the severity of confirmed COVID-19 cases was not considered, and the study was conducted before the gamma variant peaked in São Paulo. Thus, the relevant information we present here is from the Instituto Adolfo Lutz (IAL), a public health laboratory and a reference laboratory for the diagnosis and genomic surveillance of respiratory viruses, located in São Paulo City.

Using next-generation sequencing, more than 2 000 complete SARS-CoV-2 genomic sequences have been performed at IAL and submitted to GISAID, and more than 40 SARS-CoV-2 lineages were identified, including the VOCs gamma and Alpha (8). An increase in the gamma variant had been detected since January 2021 in São Paulo, and on May 21, 2021, it accounted for ˜80% of COVID-19 cases (9).

The samples received by IAL for sequencing were obtained from 17 different Regional Departments of Health under the Secretary of Health of São Paulo (*Departamento Regional de Sáude*, DRS-I to DRS-XVII) (9). These DRSs vary in geographic location and characteristics, population movement, traditions in public health policy, primary health care networks, and socioeconomic conditions. Thus, they could accurately represent the actual scenario of SARS-CoV-2 lineages that circulate in São Paulo (9). It is noteworthy, that only samples with clinical and epidemiological relevance were selected by the Epidemiological Surveillance Center of São Paulo and sent to IAL for sequencing.

At IAL, RNA samples were extracted using an automated RNA extraction procedure (kit Extracta Fast, Cod. MVXA-P016 Fast and Loccus Extracta 32 equipment, SP, Brazil), according to the manufacturer’s instructions. The cDNA was obtained using SuperScript IV VILO Master Mix (Invitrogen, USA), and entire genome sequencing was performed on an Ion Torrent S5 platform using the AmpliSeq™ SARS-CoV-2 (Thermo Fisher Scientific Inc., USA), resulting in complete sequences of SARS-CoV-2 strains without gaps. The readings were assembled with the IRMA: Iterative Refinement Meta-Assembler (CDC, USA), and the sequences were sent to the Global Initiative on Sharing Avian Influenza Data (GISAID).

The study was approved by the IAL Ethics Committee for Research CEPIAL No. 4.382.183 under the Ministry of Health protocol number CAAE–37513020.7.0000.0059. Data is presented anonymously.

This study presents data from 20 COVID-19 vaccinated individuals from São Paulo that needed hospitalization and/or died from COVID-19, for which the SARS-CoV-2 sequencing was conducted at IAL (table 1). Almost all cases were vaccinated with CoronaVac and belonged to vaccinated priority groups, such as HCWs and institutionalized elderly persons. They had the gamma variant, except one person who was infected with the Alpha variant (patient code 16). Ten patients had severe symptoms, including severe acute respiratory syndrome; almost all of them were HCWs, except for one who was an elderly woman in a nursing home (table 1, patient code 4). Samples from severe cases were sent to IAL by different DRS. On the other hand, out of ten COVID-19 deaths, six originated from DRS VI (patient codes 12 to 17). Deaths were mainly observed in the elderly at nursing homes (probably because of immunosenescence and/or lack of physical distancing), and severe cases were from younger HCWs at intensive care units or emergency rooms (74.3 years versus 54.7 years, *p* = 0.0232). Overall, after the second vaccine dose, symptoms appeared at 16–29 days.

Although all HCWs and persons over 60 years of age had been vaccinated in Sao Paulo, and institutionalized elderly persons and frontline HCWs became ill or died from COVID-19, there were no cases from the elderly living outside nursing homes or in laboratory personnel. This stresses that the former groups were at high risk for COVID-19, probably because of the high dissemination/transmission and severity of the SARS-CoV-2 gamma VOC, and the close contact between infected and non-infected individuals.

Interestingly, out of the six deaths reported in DRS VI (Bauru), two were physicians working in close contact with COVID-19 patients (patient codes 12 and 14), two lived in the same nursing home but were infected with different VOCs (patient codes 13 and 16), and two lived in the same nursing home with another patient who was still alive (patient codes 15, 17, and 4, respectively). Concerning the increased number of severe COVID-19 cases in DRS VI, the genomic surveillance of SARS-CoV-2 carried out at IAL in March 2021 showed that the new gamma variant was present in 39.5% of the sequences sent from DRS VI for analysis. This high circulation of the gamma variant in DRS VI, contrasted with the low percentages detected in other DRSs of the state at that time (10), highlighting this region as the main propagation center of the SARS-CoV-2 gamma variant of São Paulo state.

In general, data suggests that even when vaccinated, elderly persons in nursing homes and persons caring directly or in close contact with patients with the SARS-CoV-2 gamma variant can become infected and develop severe illness. Additionally, we could not exclude the possibility that these patients had been infected with the gamma variant before or near the date of their second vaccine dose. Hence, the vaccine could have shown enhanced or deregulated immune response, leading to hyper-inflammation and acute respiratory distress syndrome and/or multi-organ failure (11). Unfortunately, we did not have access to the laboratory data of these patients, and we do not know if any immunological parameters were evaluated. However, corroborating our data, the emergence of the gamma variant in Brazil has shown a higher transmission rate, high viral load, and a greater risk of severity and lethality for COVID-19 (12, 13).

**TABLE 1.** Epidemiological and virological data of COVID-19 vaccinees who developed severe symptoms or died from COVID-19 in São Paulo, Brazil

Patient code	Sex/Skin color	Age (years)	Risk factor	DRS	Vaccine	Dose		Clinical status	Symptoms onset	SARS-CoV-2 lineage	GISAID accession number
						1^st^	2^nd^				
1^a^	Male/White	40	Frontline HCW	DRS I	Pfizer/ BioNTech	Aug 15, 2020	Nov 15, 2020	Alive	Feb 21, 2021	Gamma	EPI_ISL_1219021
2^b^	Male/NI	54	Frontline HCW	DRS I	Coronavac	Feb 12, 2021	…	Alive	Mar 1, 2021	Gamma	EPI_ISL_1358287
3	Male/White	45	Frontline HCW	DRS II	CoronaVac	Jan 22, 2021	Feb 12, 2021	Alive	Mar 13, 2021	…^**d**^	…
4	Female/Black	94	Nursing home	DRS VI	CoronaVac	Jan 27, 2021	Feb 17, 2021	Alive	Mar 7, 2021	Gamma	EPI_ISL_1821208
5	Female/White	50	Frontline HCW	DRS VII	CoronaVac	Jan 18, 2021	Feb 8, 2021	Alive	Feb 28, 2021	Gamma	EPI_ISL_2003113
6	Female/NI	67	Frontline HCW	DRS VII	CoronaVac	Jan 18, 2021	Feb 8, 2021	Alive	Mar 1, 2021	Gamma	EPI_ISL_1731577
7	Male/White	54	Frontline HCW	DRS IX	CoronaVac	Jan 29, 2021	Feb 19, 2021	Alive	Feb 28, 2021	Gamma	EPI_ISL_1821210
8	Female/White	48	Frontline HCW	DRS IX	CoronaVac	Jan 29, 2021	Feb 19, 2021	Alive	Feb 28, 2021	…^**d**^	…
9	Female/White	52	Frontline HCW	DRS XIV	CoronaVac	Jan 21, 2021	Feb 11, 2021	Alive	Feb 27, 2021	Gamma	EPI_ISL_1625976
10	Male/Mulatto	43	Frontline HCW	DRS XV	CoronaVac	Jan 22, 2021	Feb 19, 2021	Alive	Mar 8, 2021	Gamma	EPI_ISL_2003112
11	Female/NI	86	Nursing home	DRS I	CoronaVac	Feb 11, 2021	Mar 4, 2021	Dead	Mar 23, 2021	Gamma	… ^**e**^
12	Male/White	83	Frontline HCW	DRS VI	CoronaVac	Jan 21, 2021	Feb 11, 2021	Dead	Mar 5, 2021	Gamma	EPI_ISL_1821207
13	Female/White	96	Nursing home	DRS VI	CoronaVac	Jan 27, 2021	Feb 17, 2021	Dead	Mar 5, 2021	Gamma	EPI_ISL_2003111
14	Male/White	66	Frontline HCW	DRS VI	CoronaVac	Jan 25, 2021	Feb 15, 2021	Dead	Mar 13, 2021	Gamma	EPI_ISL_2003148
15	Male/White	70	Nursing home	DRS VI	CoronaVac	Jan 27, 2021	Feb 17, 2021	Dead	Mar 18, 2021	Gamma	EPI_ISL_2003149
16	Female/White	88	Nursing home	DRS VI	CoronaVac	Jan 27, 2021	Feb 17, 2021	Dead	Mar 12, 2021	Alpha	EPI_ISL_2003150
17	Male/White	77	Nursing home	DRS VI	CoronaVac	Jan 27, 2021	Feb 18, 2021	Dead	Mar 12, 2021	Gamma	EPI_ISL_2003151
18	Female/White	59	Frontline HCW	DRS VII	Coronavac	Jan 21, 2021	Feb 11, 2021	Dead	Mar 12, 2021	Gamma	…^**e**^
19	Male/Yellow^f^	80	Nursing home	DRS IX	CoronaVac	Jan 22, 2021	Feb 12, 2021	Dead	Mar 15, 2021	Gamma	EPI_ISL_2003152
20^c^	Female/Mulatto	38	Frontline HCW	DRS XV	ChAdOx1	Jan 28, 2021	…	Dead	Mar 13, 2021	Gamma	EPI_ISL_1821209

***Source:*** Prepared by the authors from the results.

**Note:** NI: Not informed; HCW: Health care worker; DRS: Regional Department of Health (*Departamento Regional de Saúde*); GISAID: Global Initiative on Sharing Avian Influenza Data.

a Physician vaccinated with Pfizer/BioNTech during phase III vaccination conducted in Brazil.

b Physician from the intensive care unit, who presented symptoms of COVID-19 before receiving the second dose of Coronavac scheduled for March 5, 2021.

c Nursing technician from an emergency ward who died before receiving the second dose of ChAdOx1 vaccine scheduled for April 2021.

d Sequencing impossible (low quality of RNA sample.)

e Sequence with gaps, but confirmed as belonging to the gamma variant.

f Person of Asian descent.

Public health policy campaigns prioritizing continuous vaccination and non-pharmacological prevention measures to avoid the emergence of new variants, such as physical distancing, masks, good ventilation, avoiding crowds, and hand washing, need to continue. Supporting these needs, the delta VOC has been recently detected in Brazil, including the state of São Paulo (14). Currently, São Paulo presents another COVID-19 vaccination scenario: by August 2, 2021, 36 500 289 individuals over 28 years old were vaccinated (26 286 397 with one dose, 9 122 123 with two doses, and 1 091.769 with a single dose); ChAdOx1 accounted for 44.46%, CoronaVac for 41.02%, Pfizer/BioNTec for 11.53% and Janssen/Johnson & Johnson for 2.99% of these vaccinations (4). The government of São Paulo scheduled vaccinations for all individuals above 18 years of age by August 16, followed by adolescents, and then children. Next year, the plan includes revaccination for priority groups starting with HCWs. However, there is a lack of awareness among the Brazilian population on the use of non-pharmacological measures to prevent new COVID-19 outbreaks.

In conclusion, despite the low number of severe COVID-19 cases in vaccinated individuals reported up to May 15, 2021 to the Secretary of Health of São Paulo and sent to the IAL for SARS-CoV-2 genomic surveillance, the present data confirm high transmission rates, with an increased risk of severity and lethality of the COVID-19 gamma variant in the two high-risk groups vaccinated in São Paulo, Brazil, i.e. frontline HCWs and the elderly in nursing homes.

## Disclaimer.

Authors hold sole responsibility for the views expressed in the manuscript, which may not necessarily reflect the opinion or policy of the Pan American Journal of Public Health and/or those of the Pan American Health Organization.
